# Biomedical Evaluation of Cortisol, Cortisone, and Corticosterone along with Testosterone and Epitestosterone Applying Micellar Electrokinetic Chromatography

**DOI:** 10.1100/2012/268120

**Published:** 2012-03-12

**Authors:** Tomasz Bączek, Ilona Olędzka, Lucyna Konieczna, Piotr Kowalski, Alina Plenis

**Affiliations:** Department of Pharmaceutical Chemistry, Medical University of Gdańsk, Hallera 107, 80-416 Gdańsk, Poland

## Abstract

The validated micellar electrokinetic chromatography (MEKC) was proposed for the determination of five steroid hormones in human urine samples. That technique allowed for the separation and quantification of cortisol, cortisone, corticosterone, testosterone, and epitestosterone and was sensitive enough to detect low concentrations of these searched steroids in urine samples at the range of 2–300 ng/mL. The proposed MEKC technique with solid-phase extraction (SPE) procedure was simple, rapid, and has been successfully applied as a routine procedure to analyze steroids in human urine samples. The MEKC method offered a potential in clinical routine practice because of the short analysis time (8 min), low costs, and simultaneous analysis of five endogenous hormones. Due to its simplicity, speed, accuracy, and high recovery, the proposed method could offer a tool to determine steroid hormones as potential biomarkers in biomedical investigations, what was additionally revealed with healthy volunteers.

## 1. Introduction

Measuring steroids' profile can be suitable as a screening test indicating dysfunction of steroid-forming adrenals and gonads, for example, during the Cushing syndrome [1, 2]. However, cortisol, cortisone and corticosterone are also called as “stress hormones” and belong to the endogenous steroid hormones, playing several specific biological functions. Stress is known to induce enhanced activity of the pituitary-adrenal axis, which results in increased secretion of corticosteroids from the adrenal cortex. Therefore, the ratio between the physiologically active cortisol and its metabolite cortisone might be useful to biomedical study, because both are necessary in several metabolic processes such as adaptation to stress [3]. Abnormal metabolism of cortisol might also be related to insulin resistance [4], obesity [5], hypertension [6], type-2 diabetes mellitus [7], and apparent mineralocorticoid excess syndrome [8]. Moreover, cortisol and cortisone increase when nervous tension occurs, for example, during sport training or competition [9]. Recently, the cortisol/cortisone ratio has been proposed as a useful doping test in athletes because they illicitly use corticoids to improve their performance [10]. Cortisol may serve as a diagnostic tool to detect depressive disorders and chronic fatigue, because of its significantly higher level observed in the plasma [11] and urine [12]. Compared to healthy volunteers the urinary total cortisol/cortisone excretion ratio was enhanced in depressed patients [13]. In our previous study the plasma cortisol/cortisone ratio did not differ between depressed patients and control group [14]. Moreover, corticosterone is the glucocorticoid involved in regulation of fuel metabolism, immune reactions, and stress responses [15].

On the other hand, testosterone is an endogenous androgen having both androgenic and anabolic effects in the human body. The normal levels of testosterone and epitestosterone in the urine of healthy male volunteers are 30–60 ng/mL for both [16], while in the urine of healthy females their levels are less than 0.6 ng/mL [17]. In men, measurement of low concentrations of testosterone is needed for diagnosis and treatment of hypogonadism and to monitor androgensuppression therapy during prostate cancer treatment. In women, testosterone is measured as part of the investigation of alopecia, acne, hirsutism, osteoporosis, libido disorders, detection of androgen-secreting tumors, late-onset congenital adrenal hyperplasia (CAH), polycystic ovarian syndrome (PCOS), and other endocrine and reproductive diseases [18–20]. Moreover, the ratio of the two compounds, that is, testosterone/epitestosterone in male urine, is stable [21], and if the ratio is above 4, it has a doping effect in athletes [22]. Because of that, epitestosterone can be applied illegally to mask testosterone ingestion. In 1992 the Olympic Committee published the upper limit of the epitestosterone level in urine, set at 200 ng/mL.

Currently, capillary electrophoresis (CE) techniques are employed as major very useful separation tools for the analysis of steroids with reliable result [23]. Moreover, a kind of the CE method, called micellar electrokinetic chromatography (MEKC), has been tried [24–28]. It should be emphasized that MEKC, where the micelles are regarded a pseudostationary phase and influence largely the separation behavior of hydrophobic analytes, has been employed successfully for neutral and charged analytes ever since Terabe et al. [25] first proposed it in 1984. CE is a potent separation technique carrying advantages such as low volume of the sample, high efficiency, speed and low reagent consumption. The MEKC method can be used to detect neutral or hydrophobic analytes, if a surfactant is added to the running buffer. In the current work, sodium dodecyl sulphate (SDS) in its wide use of the anionic surfactant in the MEKC was examined, which in the effective factors improves resolution because neutral species are separated in hydrophobic interaction with the SDS micelles. As yet, there has been no published method for simultaneously determination of five steroid hormones in human urine samples.

Based on the our previous experience [26], the aim of the current study was to develop a rapid, simple and robust quantitative procedures for the simultaneous determination of cortisol, cortisone, corticosterone, testosterone and epitestosterone during one run of MEKC separation. The elaborated method has been successfully applied to quantification of five steroids, treated as biomarkers in clinical routine practice.

## 2. Materials and Methods

### 2.1. Chemicals and Reagents

Analyzed substances, cortisol (11*β*,17*α*,21-trihydroxypregn-4-ene-3,20-dione), cortisone (4-pregnene-17*α*,21-diol-3,11,20-trione), corticosterone (4-pregnene-11*β*,21-diol-3,20-dione), testosterone (17-*β*-hydroxyandrost-4-en-3-one), epitestosterone (17-*α*-hydroxyandrost-4-en-3-one), and internal standard dexamethasone (9*α*-fluoro-11*β*,17*α*,21-trihydroxy-16*α*-methylpregn-1,4-dien-3,20-dione) were purchased from Sigma (St. Louis, MO, USA). Stock solution of analyzed substances was prepared by accurately weighing 10 mg of each hormone in 10 mL of methanol. The working solutions were also prepared just before use by diluting the stock solutions as appropriate with triple distilled water. They were stored in the dark under refrigeration to avoid possible decomposition. Reagents used for the preparation of samples and buffer solution such as sodium hydroxide, hydrochloric acid, sodium tetraborate decahydrate, and ammonium hydroxide were delivered by POCh (Gliwice, Poland). Sodium dodecyl sulfate (SDS), dichloromethane, acetone, HPLC grade acetonitrile, and methanol were supplied by Merck (Darmstadt, Germany). All chemicals were of analytical grade and applied without further purification. Highly pure water was obtained from Milli-Q equipment (Millipore, Bedford, MA, USA). Separation of compounds of interest before MEKC assay was preceded by sample preparation with solid-phase extraction (SPE) using hydrophilic-lipophilic balance (HLB) cartridges (200 mg, 6 mL), which were purchased from Supelco (Park Bellefonte, PA, USA).

### 2.2. Analytical Conditions for MEKC

The experiments in steroid separation were performed using a capillary electrophoretic system (P/ACE 2100, Beckman Instruments, Fullerton, CA, USA) equipped with an automatic injector, a filter-carrousel UV detector, a temperature control device, and a data acquisition system supplied by the manufacturer (Beckmann P/ACE System Gold Software). Electrophoretic separation was carried out using a fused silica capillary with the i.d. of 75 *μ*m, total length of 57 cm (effective length of 51 cm), at the constant voltage of 17 kV, and the temperature of 22°C (±0.1). The UV detection for steroid analysis was set at the wavelength of 254 nm. In order to achieve good repeatability of the migration time and to avoid solute adsorption on the capillary wall, the following washing procedure was performed. Before the first use, the capillary was conditioned by flushing (20 psi) with 1 M NaOH solution for 10 min, then with deionized water for 10 min. At the beginning of each day, the capillary was rinsed with 0.1 M NaOH (regeneration solution), deionized water, and finally with the running buffer for 10 min. Because sample components can be adsorbed on the capillary surface and change the effective charge on the wall, the capillary was flushed with methanol (0.5 min) after each injection. In order to provide good reproducibility, the capillary was also reconditioned between the runs with the regeneration solution (1 min) and deionized water (1 min). The analytes were loaded into the capillary at the anode for 2 s in an argon injection, at the pressure of 0.5 psi, while the detector was placed on the cathode end of the capillary. The separation of the analyzed endogenous steroid by the MEKC method was performed using the running buffer consisting of 20 mM sodium tetraborate (pH = 9.3) and 25 mM SDS, the proportion optimized in earlier experiments. Peak identification was conducted by spiking with the analyte to be identified.

### 2.3. Sample Preparation

Steroids occur in human urine specimens in a conjugated and nonconjugated (free) form. Depending on the relationship between the two forms, a small part of the steroid hormones in urine are found in the free form (below 1%), while the major fraction is contained in glucuronide and sulphate conjugates (99%). To determine the total steroid level, it was necessary to include the hydrolysis step. However, we were aware that if urine samples were taken at random, for example, after or during a stressful situation, the creatinine level would require determination. Therefore, the steroid concentration level was corrected resulting in the steroid-to-creatinine ratio. The creatinine level was evaluated using a diagnostic kit based on the colorimetric method (PZ Cormay, Lublin, Poland). This made the results of the normalized steroid measurements realistic. The urine samples were obtained from adult healthy volunteers between 7.00 and 9.00 AM. All samples were frozen at −20°C until the analysis. Before extraction, the samples were brought to the room temperature and an acid hydrolysis in water bath with 36% hydrochloric acid was performed for 1 hour at 95°C. After the hydrolysis, the urine samples were brought back again to the ambient temperature and subjected to a further extraction process. Before electrophoretic separation the calibration urine specimens (15 mL in volume), with various concentrations of the analytes ranging from 2 to 300 ng/mL and with the internal standard solutions to achieve the final concentrations of 200 ng/mL of dexamethasone, were spiked. SPE was chosen for effective and high recovery of the steroids in the HLB column (6 mL, 200 mg). Urine samples were prepared as detailed above then extracted in several steps. First, the HLB cartridges were conditioned with 5 mL of methanol followed by 10 mL of deionized water. Then, the steroid-spiked urine samples or samples obtained from volunteers were carried to the SPE sorbent. Next, each sample was slowly passed through the SPE column using the Baker system under the vacuum conditions. Once the samples were washed with 4 mL of an acetone-water mixture at the ratio of 25 : 75 (*v/v*), then, with 2 mL of 2% ammonium hydroxide in 50% methanol followed by 2 mL of distilled water in order to obtain the final steroid elution, we applied 4 mL of methanol in two steps. The organic solvent was evaporated to dryness at 45°C in a water bath then reconstituted with 100 *μ*L 2 mM sodium tetraborate decahydrate. The samples were centrifuged for 7 min at 3000 rpm and injected into the CE system. The same sample preparation was applied to the urine obtained from volunteers.

## 3. Results and Discussion

### 3.1. Optimization of Sample Extraction

#### 3.1.1. Hydrolysis Procedure

Acidic hydrolysis was performed before extraction procedure. For that purpose a mineral acid was added to urine to cleave steroids from the conjugation form. We realize that the yield of hydrolysis is strongly influenced by several parameters: the acid concentration, temperature, and time of reaction. In our experience, using high temperature for a relatively short time (1 hour), under strictly controlled conditions, did not cause any significant disruption of the steroids. Moreover, complete hydrolysis was achieved by placement of urine samples using 36% hydrochloric acid under a stream of compressed air in water bath closed with a tight lid at the temperature of 95°C.

#### 3.1.2. Extraction Procedure

In order to develop the best sample preparation procedure, the SPE and liquid-liquid extraction (LLE) were tested. At first, LLE was employed, but many problems were encountered because of the emulsion formation. Moreover, the extraction recovery was not satisfactory, and sample loss during the process was observed. The serious limitations of that particular extraction method come down to the fact that it is a time-consuming process involving high consumption of organic solvents, all of which make the method unfriendly in terms of either the environmental or human health protection. Next, solid-phase extraction (SPE) procedures with silica-based apolar columns including C_18_, C_8_, CN, functionalities, and hydrophilic-lipophilic balance (HLB) cartridges were tested. To that aim blank urine samples were spiked with 10, 50, and 100 ng/mL of steroids, and different organic solvents were tested. The extraction efficiency was acceptable when methanol as eluent and an HLB column were used, yielding high recoveries of 95.8% for 10 ng/mL, 94.6% for 50 ng/mL, and 92.1% for 100 ng/mL (Table 1). Thanks to the HLB cartridges, the extraction process can be made more precise and less tedious than in the case of a normal SPE column, and lead to more effective extraction with higher recovery. Additionally, the intense, dark color the urine samples turned to after acid hydrolysis used to disturb extraction process and decrease its efficiency.

A mixture of acetone and water (25 : 75, *v/v*) eliminates the arduous pigment. Hence, another step in the extraction process was added, namely, the stage of washing with 2% ammonium hydroxide in 50% methanol so as to get rid of the dark color of the urine samples. Next, we tested further modifications of the pH of the organic eluent. The best efficiency of steroid extraction was noticed at pH close to 7.0, where no interaction between the silica sorbent and the analytes occurs. The SPE was performed using the automated Baker system (Darmstadt, Germany), which improves accuracy and precision of the results and improves the laboratory productivity. Compared to the conventional LLE, the applied SPE procedure was environmentally friendly and consumed low volumes of organic and hydrophilic solvents.

#### 3.1.3. Effect of Running Buffer and SDS on Separation

Since to steroid hormones are electrically neutral molecules of low hydrophobic (in free form) or hydrophilic (in the form of conjugates) properties, the choice of the running buffer significantly impacts on the resolution of the analytes. The separation principle of the capillary zone electrophoresis (CZE) mode is based on the differences between compounds in terms of their electrophoretic mobility. Unfortunately, the electrophoretic mobility of some steroids is very similar. In effect, the traditional CZE method using borate or phosphate electrolyte only will result in nonseparation. That is why MEKC with SDS, as the most common anionic surfactant at a concentration, greater than its critical micelle concentration is added to the buffer solution and was successfully employed in our study. The SDS micelles enhanced solubility of the analytes and offered excellent resolution. This can be accompanied by differential partitioning of the analytes between the micellar and aqueous phases. The influence of the SDS concentration in the optimized tetraborate buffer was further evaluated for the resolution of all investigated steroids. The concentration of the micelle-forming agent (SDS) was tested from 10 to 50 mM. Raising the concentration of SDS in the running buffer resulted in an increase of the migration times for the compounds of interest. Experimental results indicated that the SDS concentration of 25 mM contributed significantly to the peak quality and migration time and could yield complete baseline separation for all steroids. Thus, the concentration of 25 mM SDS and 20 mM tetraborate in the buffer at pH 9.3 was selected to obtain a good peak shape, low peak width, short migration time, and higher efficiency. Furthermore, the chosen separation conditions eliminated many of the potential interferences, including those ones coming from most endogenous substances of urine biological sample.

### 3.2. Optimization of Instrument Parameters

It was interesting to study the parameters significantly influencing electrophoretic separation, for example, voltage, injection time, and temperature, in the achievement of good resolution, symmetry, and high peaks of the steroids. To identify the suitable voltage to be applied, its effect was studied within the range of 10–30 kV. Under the described conditions (20 mM tetraborate buffer at pH 9.3 and 25 mM SDS), increased applied voltage shortened the analysis times and sharpened the peaks. Higher separation voltages not only increased the electrophoretic velocity of the analytes, but also increased the current and the Joule heat. This can be mitigated by lowering the ionic strength of the running buffer or by enhancing heat dissipation. Therefore, in order to limit heating inside the capillary, the maximum applied voltage was chosen based on the Ohm's plot (current versus voltage) and the voltage of 17 kV was finally used.

Moreover, changes of the capillary temperature can cause variation in efficiency, viscosity, electrophoretic mobility, and the migration times of the analytes. The effect of the capillary temperature on selectivity and the migration time was examined over the range of 18–25°C. The best conditions giving sufficient resolution and a good level of baseline noise were achieved at the temperature of 22°C (±0.1). Optimized hydrodynamic injection was employed to introduce the samples into the capillary. The urine extracts were analyzed by MEKC using the injection time of 2, 5, and 7 s. It was observed that the 2 s injection (corresponding approximately to the 8 nL injection volume of the sample) led to achieving the most efficient separation of all steroids. The longer injection times resulted in broadened, overlapping peaks.

### 3.3. Validation of the Method

The calibration samples were prepared and quality control set by spiking the steroid-free urine samples (charcoal-stripped) with a steroid spiking solution to obtain the final concentrations of 2, 10, 50, 100, 200, 300 ng/mL.

The linearity of the calibration curve was determined for the range of 2 to 300 ng/mL and evaluated by analyzing six different concentrations of steroids. The calibration curve was constructed by plotting the ratios of the peak height against the corresponding concentrations. Each concentration was injected in six replicates. The following regression equations were calculated, including the slopes, the intercepts, and the correlation coefficients, as listed in Table 2. To determine the limit of detection (LOD), ten independent blank urine extracts were spiked with suitable steroid concentrations, injected, and measured after the extraction procedure. The LOD was expressed as the analyte concentration corresponding with the peak expressed as the signal-to-noise ratio of 3. The limit of quantitation (LOQ) was expressed as the analyte concentration corresponding with the peak and expressed as the signal-to-noise ratio of 10. The LODs and LOQs were established at 0.5 and 2 ng/mL, respectively.

**Figure 1:**
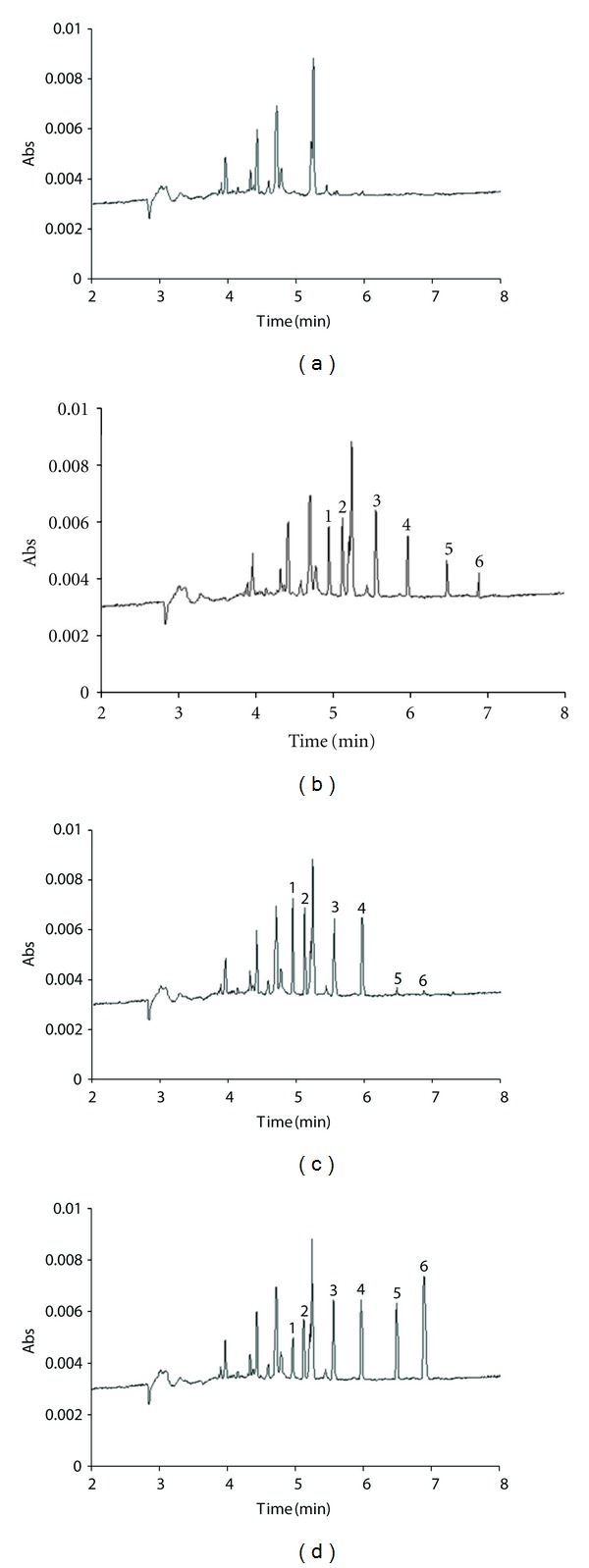
Typical electropherograms obtained for (a) steroid-free urine sample; (b) male urine sample; (c) female urine sample; (d) urine sample spiked with 50 ng/mL of cortisol (1), 50 ng/mL of cortisone (2), 200 ng/mL of dexamethasone (3) (I.S.), 100 ng/mL of corticosterone (4), 100 ng/mL of testosterone (5), and 100 ng/mL of epitestosterone (6). Conditions of electrophoretic separation: 17 kV, injection 2 s, UV, *λ* = 254 nm, running electrolyte: 20 mM Na_2_B_4_O_7_, 25 mM SDS.

The specificity of the method was assessed by conducting a comparative analysis of blank urine samples and urine samples spiked with steroids after the extraction procedure earlier described in this paper. The representative electropherograms are presented in Figures 1(a) and 1(b). No interferences were observed during the electrophoretic runs of the urine samples in the area where steroid or internal standard peaks appear. The specificity of method was further confirmed by identification of the steroid and internal standard peaks based on the migration times as well as on the UV spectrum. Finally, urine samples spiked at three chosen concentration levels (50, 100, and 200 ng/mL for cortisol, cortisone, and corticosterone; 20, 50, and 100 for testosterone and epitestosterone, resp.) were analyzed in a six-replicate analysis of the samples on the same day (repeatability) and in triplicate between days (reproducibility). The values of repeatability and reproducibility ranged between 0.19 and 1.32%, and between 0.14 and 8.34%. The intra- and inter-day accuracy of the assay was tested by fortifying the mixture of the steroid solution in three concentrations with steroid-free urine sample and determining the percent recovery of steroids (identified concentration/nominal concentration) × (100%). The intra- and interday accuracy stayed above 92.0 and 87.3%, respectively. Absolute recovery was evaluated by comparing the peak height for the urine samples, obtained after extraction with the height obtained for nonextracted samples containing the same concentration of the analyzed steroids. Absolute recovery was determined for three concentration levels (*n* = 6). The mean absolute recoveries of the analyzed hormones were above 91.2%. As a part of the validation process, the freeze-thaw stability of each steroid in human urine was also tested by determining three replicates at three concentration levels after three cycles of freezing (−20°C) and thawing (room temperature) at the interval of two months. The assay was based on back-calculated concentrations. The obtained data indicated that steroids in urine samples remained stable when stored at the temperature of −20°C.

### 3.4. Application to the Real Urine Samples

Success of any analytical method comprising also CE-based one can be proven when the number of real-world applications increases. The number of CE applications is observed as to be growing, just as its reoccurrence in the reviews published recently and based on clinical, forensic, and biomedical applications.

In the last years many authors demonstrated the application of their methodology developed by the determination of steroid hormones in the analysis of real samples from people [23]. Unfortunately, often without giving details on, for example, how large the groups of healthy volunteers were studied, physical data of patients such as height, weight, creatinine clearance described, as well as finally results on the steroid hormone levels in the analyzed groups. Moreover, recommendations on quantitative methods to assess hormonal diseases are often proposed; however no direct confirmations on usefulness of these methods treated as biomedical applications in real human group can be easily found in the literature [23].

In the current study, under the optimized experiment conditions, the quantitative evaluation of the endogenous steroid level was carried out using the developed MEKC method. Investigations were performed in compliance with the rules set by the ethics committee, and the study protocol was also approved by the Ethical Committee of the Medical University of Gdańsk, Poland.

All participating volunteers (10 males and 10 females) represented the average age of 23 ± 2.6 years, the body weight of 67 ± 13 kg, and the height of 171 ± 9 cm.

For the evaluation of glucocorticoid concentrations as biomarkers of stress the urine samples should be collected as soon as possible after stress situation. On the other hand, the levels of urinary glucocorticoids could be changed due to the density of this fluid. Because it was confirmed that creatinine, being a product of muscle metabolism, is normally lost in the urine at a relatively steady state, the ratio of urinary corticosteroids to creatinine should be used to gain a correct urinary concentration of compounds of interest. Moreover, abnormal physiological concentrations of creatinine in urine samples may signal renal failure and/or a reduced glomerular filtration. It may cause increasing or decreasing amounts of analyzed hormones in urine. The mean levels of creatinine in urine were between 0.88 and 2.39 mg/dL. These results also confirmed that no participation possessed a dysfunction of kidney. Next, the cortisol, cortisone, and corticosterone levels in all urine samples were determined.

The average total concentrations of cortisol, cortisone, and corticosterone in the urine samples obtained from the searched healthy volunteers were 115.80 ± 49.45 ng/mL, 265.04 ± 141.07, and 64.29 ± 29.65 ng/mL, respectively. The urinary total excretion ranged from 41.67 ng/mL to 193.33 ng/mL for cortisol and from 55.00 ng/mL to 553.50 ng/mL for cortisone, and the urinary total corticosterone was recorded between 24.43 ng/mL and 127.14 ng/mL. The overall average concentration of the urinary steroids is shown in Table 3. The concentration of urinary cortisol was found remaining within the physiological range in most volunteers, though in a few samples the level was identified at the higher end of the normal range of the values reported as the reference ones (50–250 ng/mL). The same volunteers were also found to have increased levels of the other stress hormones (cortisone and corticosterone). For example, one can find such a situation in the case of volunteers nos. 8, 10, and 13.

Next, the determination of urinary cortisol/cortisone ratio was used to assess the renal activity of 11 *β*-hydroxysteroid dehydrogenase type 2, the enzyme which converts biologically active cortisol to inactive cortisone and which is essential for maintaining water balance in the body by controlling the levels of cortisol. A decrease of its activity (e.g., through disease or inhibition) leads to an increase in the level of cortisol in the body. The values obtained in the current experiment using the searched healthy volunteers ranged from 0.11 to 1.54 and that did not indicate a metabolic disorder or doping. It can be seen that the urinary cortisol/cortisone ratio could be a valuable parameter to clinicians as an early predicator for the onset of hypertension.

In the case of androgens, the average concentration levels of the endogenous testosterone and epitestosterone were 10.77 ng/mL ± 10.76 and 7.78 ± 9.06 ng/mL, respectively. The urinary total testosterone excretion ranged from 2.6 to 38.00 ng/mL, and the urinary total epitestosterone was recorded between 2.17 ng/mL and 30.83 ng/mL.

An important parameter in terms of doping control is also testosterone/epitestosterone ratio. The norm for urinary testosterone/epitestosterone ratio is below 4. Usually, the amounts of testosterone and epitestosterone are in similar proportions and their normal presence in urine has been found, on an average to be roughly equal to 1 : 1, although it varies on a case-specific basis. Physical effort does not modify this ratio but it can be increased by the use of doping agents. The values of testosterone/epitestosterone ratio obtained in the current experiment from the searched healthy volunteers ranged from 1.1 to 3.6 and no doping with testosterone in the study group could be identified.

## 4. Concluding Remarks

The developed and applied novel micellar electrokinetic chromatography method (MEKC) proved to be efficient and allowed for the simultaneous determination of five steroids of interest over a short total time of 8 min.

Full automation, high efficiency, rapidness, low solvent consumption, and low costs made the proposed MEKC method attractive, especially in terms of suitability for routine analysis during clinical investigations. The method presented in this paper appeared to be properly optimized and fully validated, including the proved specificity, linearity, sensitivity, precision, and accuracy. By using the HLB columns for the SPE during the sample treatment procedure, and by an optimization of separation conditions, it was possible to achieve satisfactory detection and quantification limit. The described MEKC method has been successfully applied during the determination of endogenous steroid profiles in human urine obtained from healthy volunteers. The assessment of the proposed methods in terms of detection and evaluation of the steroid level obtained from a real human group of 20 healthy volunteers proved its usefulness in the medical diagnostic practice and made it recommendable for biomedical investigations.

## Figures and Tables

**Table 1:** Analytical extraction efficiency test of the analyzed steroids after various extraction columns and solvents.

Column extraction	Concentration added (ng/mL)	Dichloromethane			Methanol		
		Concentration found (ng/mL) (*n* = 3) (mean ± SD)	Recovery (%)	RSD (%)	Concentration found (ng/mL) (*n* = 3) (mean ± SD)	Recovery (%)	RSD (%)
C8	10	5.5 ± 0.7	54.6	12.9	6.2 ± 0.7	62.3	11.6
	50	53.1 ± 6.1	53.1	11.4	63.4 ± 6.5	63.4	10.3
	200	111.4 ± 10.9	55.7	9.8	123.4 ± 12.2	61.7	9.9

C18	10	8.8 ± 0.6	88.4	6.6	9.1 ± 0.6	90.9	6.4
	50	42.5 ± 3.5	85.1	8.2	44.7 ± 3.2	89.4	7.1
	100	81.7 ± 5.9	81.7	7.3	89.8 ± 6.2	89.8	6.9

CN	10	3.4 ± 0.3	33.7	8.8	3.6 ± 0.3	35.6	7.6
	50	15.6 ± 1.7	31.2	10.7	17.1 ± 1.4	34.2	8.2
	100	30.8 ± 2.1	30.8	6.9	36.3 ± 3.5	36.3	9.7

HLB	10	8.9 ± 0.2	89.6	2.2	9.6 ± 0.2	95.8	2.3
	50	45.2 ± 2.4	90.4	5.4	47.3 ± 2.0	94.6	4.2
	100	92.1 ± 4.5	92.1	4.9	46.1 ± 1.8	92.1	3.9

**Table 2:** Results of regression model for the analyzed steroids.

	Total cortisol	Total cortisone	Total corticosterone	Total testosterone	Total epitestosterone
Linearity range (ng/mL)	2–300				
Slope ± SD	0.0062 ± 0.00013	0.0044 ± 0.00004	0.0077 ± 0.00008	0.0056 ± 0.00007	0.0067 ± 0.00005
Intercept ± SD	0.219 ± 0.0198	0.306 ± 0.0060	0.192 ± 0.0120	0.151 ± 0.0107	0.116 ± 0.0083
Correlation coefficient (*r*)	0.9991	0.9998	0.9997	0.9997	0.9998
*n*	6				
LOD (ng/mL)	0.5				
LOQ (ng/mL)	2.0				
Total separation time (min)	8.0				
	Migration time (min)				
Cortisol	4.95				
Cortisone	5.12				
Corticosterone	5.97				
Dexamethasone I.S.	5.55				
Testosterone	6.48				
Epitestosterone	6.66				

**Table 3:** Results of the urinary total steroid levels in healthy volunteers.

Volunteer no.	Sex	Age	Height (cm)	Body mass (kg)	Creatinine level (mg/dL)	Total level (ng/mL)				
						1	2	3	4	5
1	F	23	163	57	1.98	46.33	121.00	73.00	Nd	Nd
2	M	21	192	93	1.04	84.83	55.00	30.01	4.2	2.17
3	M	22	182	78	1.28	121.83	213.50	81.14	4.9	3.33
4	F	22	166	57	1.42	168.50	296.00	76.86	13.0	Nd
5	F	23	164	50	0.99	121.83	321.00	36.86	Nd	Nd
6	F	21	158	64	1.34	167.00	279.00	70.3	6.8	Nd
7	F	22	160	58	2.36	121.83	321.00	36.86	2.6	Nd
8	M	28	176	74	1.45	163.50	373.50	82.57	4.5	3.83
9	M	21	182	86	1.49	52.83	55.75	68.57	34.4	18.67
10	M	28	186	95	0.88	121.83	388.50	32.00	4.7	2.30
11	F	21	164	59	1.09	48.50	446.00	61.14	Nd	Nd
12	F	30	168	54	2.39	158.50	398.50	69.71	10.6	7.83
13	F	24	163	56	1.14	193.33	553.50	51.00	Nd	Nd
14	F	22	166	59	2.09	52.333	74.25	60.86	38.0	30.83
15	F	24	168	55	1.64	41.67	144.50	127.14	6.6	Nd
16	M	23	174	78	2.06	94.17	235.50	35.43	9.0	8.17
17	M	22	168	69	1.14	86.33	82.50	24.43	6.4	2.83
18	M	23	170	67	1.46	138.50	363.50	40.43	4.4	2.50
19	M	21	172	63	0.97	172.17	246.00	112.57	11.4	3.17
20	M	21	168	59	1.43	160.17	332.25	115.00	Nd	Nd
Average						**115.88**	**265.89**	**65.57**	**7.96**	**4.11**
SD						**49.53**	**141.41**	**37.87**	**12.26**	**7.75**

1: cortisol; 2: cortisone; 3: corticosterone; 4: testosterone; 5: epitestosterone; Nd: not determined.
